# Comparison of Controller Logics for Automating Vasopressor Administration Using a Hardware-in-Loop Test Platform

**DOI:** 10.3390/bioengineering13040454

**Published:** 2026-04-13

**Authors:** Michael D. Lopez, Jonathan Marrero Bermudez, David Berard, Lawrence Holland, Austin J. Ruiz, Jose M. Gonzalez, Sofia I. Hernandez Torres, Eric J. Snider

**Affiliations:** 1 Organ Support & Automation Technologies Group, U.S. Army Institute of Surgical Research, Joint Base San Antonio, Fort Sam Houston, TX 78234, USA; 2 Department of Surgery, Long School Medicine, UT Health San Antonio, San Antonio, TX 78229, USA

**Keywords:** closed-loop, automation, hemorrhagic shock, fluid resuscitation, controller development, hardware-in-loop, vasoactivity, fuzzy logic, combat casualty care

## Abstract

Hemorrhagic shock remains one of the leading causes of preventable death for both civilian and military trauma. Fluid resuscitation is the primary treatment but requires constant monitoring, particularly for volume non-responsive patients susceptible to fluid overload, pulmonary edema, and other life-threatening conditions. To overcome fluid non-responsiveness, vasoactive drugs or vasopressors can be necessary adjuvants to fluid therapy but require tedious titrations that can be difficult to manage during mass-casualty situations. This study developed and evaluated automated closed-loop vasopressor controllers for hemorrhage scenarios. Ten physiological closed-loop controller (PCLC) configurations with different underlying functionalities were tuned to be either more aggressive or conservative to reach the target mean arterial pressure. A hardware-in-loop test platform with fluid-pressure responsiveness, derived from animal data, tested each controller across three different starting pressure scenarios. The platform successfully differentiated controller designs based on performance metrics. While some configurations overshot the target and others could not reach the target pressure, strong-performing PCLCs consistently reached and maintained the target quickly. Three candidate PCLCs outperformed the rest and will be evaluated across wider scenarios to develop a robust controller design. This work accelerates PCLC-driven vasopressor administration development, providing a necessary fluid resuscitation adjuvant for precise hemodynamic management in hemorrhagic trauma.

## 1. Introduction

The optimization of pre-hospital treatment requires the adoption of novel approaches for delivering medical care for improved patient outcomes. Automation of clinical treatments is an increasingly researched approach for improving patient outcomes while reducing medical-provider cognitive burden in pre-hospital settings [1,2,3]. Physiological closed-loop controllers (PCLCs) are a potential solution to automate various health applications, such as the adoption of PCLCs in the automation of portable ventilators [4,5]. Additionally, PCLCs have been utilized to automate the process of fluid resuscitation to improve mortality outcomes for hemorrhagic shock [6,7]. PCLCs for fluid resuscitation are responsible for the automation of blood product and/or crystalloid infusions to bring a patient’s mean arterial pressure (MAP) up to a target blood pressure. This gives rise to a question about what happens when a patient becomes un-responsive to the amount of infusate they are receiving. In some clinical situations, patients who are less responsive or non-responsive to transfusion during hypovolemic shock are often treated using vasopressor drugs [8,9]. Vasopressors constrict blood vessels as another method to raise MAP to reach a target pressure. However, in the administration of vasopressors, it can be challenging to effectively titrate dosing, and mortality can still occur even as patients are receiving the vasopressor support [10]. Excess vasopressor therapy dosing has been associated with high mortality outcomes [11]. Furthermore, the lack of personnel to manage the use of vasopressors can amplify these challenges [12]. As such, the development of PCLCs for vasopressor therapy may provide insights into automation strategies under resource-limited conditions in simulation.

PCLCs for vasopressor management have been developed for certain clinical applications to be able to mitigate the issues currently associated with vasopressor management. Controllers developed for this use focus primarily on an input blood pressure signal, such as MAP, driving systems to a target value for the physiological signal. The measured input variable is fed into a controller that uses various controller architectures to reduce the error between the measured MAP and the target MAP. Many controllers use conventional control theory approaches such as fuzzy logic, Proportional–Integral (PI), and Proportional–Integral–Derivative (PID) to name a few. Rinehart et al. has developed a controller which uses a combination of a PID and a rules engine to control the infusion rate of the vasopressor based on MAP [13]. Another control architecture, anti-windup PI with an internal model controller (IMC) by Wassar et al., also uses MAP as the input/target variable. The anti-windup stops integrating the error when the vasopressor infusion rate is met and the IMC adjusts real-time drug sensitivity for improved performance and stabilization across different vasopressor responses [14]. Luspay et al. made use of a hybrid multiple-model extended Kalman filter (MMEKF) and Linear Parameter-Varying (LPV) controller setup. Their MMEKF-LPV setup, like previous controllers, uses the MAP as the target and the input variable, with the controller adjusting vasopressor dosage to regulate the blood pressure. The blood pressure is regulated using dynamic weights that change based on parameters [15]. More recent approaches by Coeckelenbergh et al. and Joosten et al. furthered the MAP-targeting vasopressor control approach by incorporating it into studies in stable clinical environments, including post-surgical intensive care unit patients following high-risk abdominal surgery and severe-brain-injury patients, where closed-loop vasopressor systems similarly demonstrated proficient time-in-target MAP performance compared to manual titration [16,17]. While many controllers exist to reach a target MAP, few explore weaning off vasopressor infusion, an important part of vasopressor management. Merouani et al. included the weaning-off logic, and this control functionality was done using a fuzzy logic architecture. This controller used MAP as both the input and target variable and worked by adjusting the vasopressor infusion rate every seven minutes to wean off of the vasopressor being administered [18]. However, nearly all prior work on PCLCs for vasopressor hemorrhage therapy was focused on a surgical-support clinical application, where a more stable patient is being managed with more extensive medical resources available compared to a hemorrhagic shock trauma situation, leaving the fluid non-responsive trauma casualty largely unaddressed in existing controller designs. Beyond vasopressor-specific controllers, the broader field of physiological closed-loop control has seen rapid diversification of controller architectures for automated drug delivery. Intelligent fuzzy controllers for intensive care drug dosage optimization have shown that integrating adaptive tuning mechanisms with rule-based fuzzy logic can manage the nonlinearities and inter-patient variability that challenge fixed-gain controllers [19]. In the domain of automated insulin delivery, Zhao et al. applied deep reinforcement learning with a dual safety mechanism for glucose regulation—an example of how data-driven methods paired with safeguard logic can balance controller aggressiveness against patient safety constraints [20]. These parallel efforts across multiple drug delivery applications point to both the need and opportunity for systematic comparison of controller architectures, as no single approach has proven universally superior for all physiological control problems.

Here, we aim to develop a vasopressor adaptive resuscitation controller (V-ARC) for simulated hemorrhagic shock resuscitation that is able to constantly monitor and make swift decisions to ensure the target MAP is met using an appropriate dosage of vasopressors. We evaluated various V-ARCs with traditional and custom control system designs on a hardware-in-loop testbed previously developed [21] and provide an analysis of the outcomes to identify the best-performing PCLC designs. In summary, this work provided the following contributions:

• Development of V-ARC, a vasopressor adaptive resuscitation controller specifically designed for hemorrhagic shock trauma scenarios rather than surgical support applications;
• Systematic comparison of traditional and custom control architectures for vasopressor management on a hardware-in-loop testbed;
• Identification of the best-performing controller logic for managing fluid non-responsiveness in a hardware-in-loop simulation.

## 2. Materials and Methods

The study design presented in this section begins with an overview of the animal study that supplied preliminary data and an explanation of the scheme used for informing PCLC tuning, followed by descriptions of each main controller type and their respective tuning processes. Next, the test platform is introduced along with the simulated patient scenarios used for performance testing. Concluding the section is a summary of the standard, modified, and aggregated performance metrics used for relative ranking of the PCLC designs.

### 2.1. Overview of Animal Study

The Institutional Animal Care and Use Committee (IACUC) at the United States Army Institute of Surgical Research approved all research conducted for this study. This proof-of-concept animal study exposed swine (sus scrofa domestica) to hemorrhagic shock and performed fluid resuscitation, as has been described previously [6]. A total of *N* = 10 female Yorkshire crossbred swine were included in this study, approximately four months in age and an average of 40 kg in weight. All animals were maintained at a surgical plane of anesthesia throughout the study, first by continuous inhalation of isoflurane (0–5%), followed by total intravenous anesthesia (TIVA) using ketamine (0–10 mg/kg/h) and midazolam (0–2 mg/kg/h); buprenorphine SR (0.24 mg/kg) was given once for analgesia.

For this study, the data used was captured during infusion of norepinephrine vasopressor (dosage shown on Table 1) from a state of hemorrhagic shock. This occurred after an initial hemorrhage to 35 mmHg and resuscitation with whole blood using an adaptive resuscitation controller (ARC) to return animals to a target MAP, which was based on the starting blood pressure of the swine prior to hemorrhage [7]. ARC maintained swine at target MAP for one hour, followed by a secondary hemorrhage event to 35 mmHg that was held for at least 10 min. Next, norepinephrine (NE) infusion alongside a constant 10 mL/min crystalloid infusion rate was started. Since a single concentration of NE solution (4.0 mcg/mL) was used, the delivered dosage was solely dependent on the infusion rate in mL/min and was how “dosage” was defined throughout controller development. Dosing was incrementally increased after MAP stabilized at each dose until reaching the target MAP. At this point, NE dosing was reduced by one step in the dosing table to observe the effect on MAP. After allowing MAP to stabilize, swine were humanely euthanized.

The data captured from this animal study were used to incorporate techniques and approaches for developing the Adaptive Neural Fuzzy Inference System (AN-FIS), step fuzzy inference system (Step-FIS), and patient-following controller (PFC) described in more detail below.

### 2.2. Overview of V-ARC Test Platform

**Figure 1:**
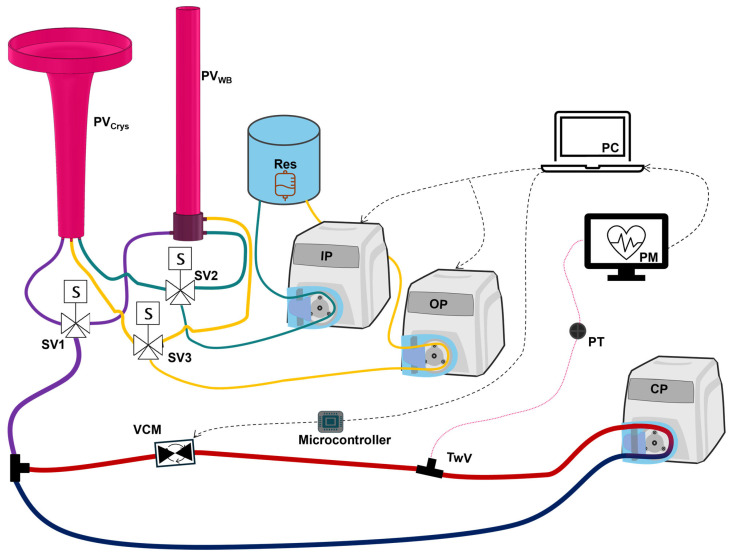
Diagram of flow loop system modified for testing automated vasopressor controllers. Diagram modified from Snider et al. [22].

For testing, the hardware-in-loop automated testbed for resuscitation controllers (HATRC) with an integrated Vasopressor Control Module (VCM) was used (Figure 1). This system and its development using large-animal study datasets have been described previously [22,23], as well as its successful use to tune hemorrhage resuscitation controllers [6,7]. Briefly, a circulating fluid loop with a peristaltic pump-driven (MasterFlex, Gelsenkirchen, Germany) pulsatile flow produces a pressure signal that is detected via arterial line by a patient monitor (Drager, Lübeck, Germany). This pulsatile pressure signal is used to calculate the MAP which can also be down-sampled, reflecting constraints such as relying on non-invasive blood pressure measurements [24], and the system can operate at physiologically relevant pressures for hemorrhagic shock resuscitation. Additional pressure disturbances and noise can be simulated by the platform as well, though noise profiles specific to the desired setting need to be characterized. Fluid reservoirs, called PhysioVessels, provide hydrostatic pressure to the system that is responsive to changes in volume. The PhysioVessels were developed based on pressure response to fluid volume data from animals to simulate the effects of fluid resuscitation on the system at an MAP level. Two additional pumps are plumbed directly to the PhysioVessels, allowing addition or removal of fluid from the system without disrupting the dynamic pressure signal from the circulating portion of the loop. The integrated VCM provides a way for system pressure (i.e., MAP) to be affected independently of volume and was designed to mimic four characteristic features extracted from in vivo MAP signals during vasopressor dosing changes, including Lag Time, the time delay from dosage change to pressure response; overshoot, how much the pressure peaks before settling; Real Response, the steady-state pressure difference; and responsiveness, the time-rate of change in pressure during the initial rise. This module comprises a stepper motor-driven needle valve and a microcontroller. When a vasopressor dosage is administered, the microcontroller calculates the anticipated pressure change, determines the required amount of occlusion by the needle valve to induce said pressure change, and actuates the motor until the required position is reached. The functions used for these calculations and the characterization of the module are further detailed in previous work [21].

### 2.3. Physiological Closed-Loop Controller Development

The five controller architectures evaluated in this study were selected to span the range of approaches used or proposed for automated drug delivery in the literature, from well-established methods to data-driven and hybrid designs. PID control was included as a standard reference benchmark, given its long history in control systems and more recent multi-drug hemodynamic management systems [25]. While PID remains widely used due to its simplicity and well-understood tuning procedures, prior work in our group on the adaptive resuscitation controller (ARC) for whole-blood and crystalloid fluid resuscitation demonstrated that fuzzy logic-based designs could match or outperform PID in hemorrhage scenarios where nonlinear patient responses and variable drug sensitivity complicated linear control assumptions [6,7]. This motivated the inclusion of two fuzzy-based architectures: Step-FIS, a custom rule-based Sugeno controller paired with adaptive step logic, and AN-FIS, which uses the learning capabilities of neural networks to tune fuzzy rule parameters from animal study data [22]. The choice to include both a manually designed (Step-FIS) and a data-trained (AN-FIS) fuzzy architecture allowed us to compare expert knowledge-driven design against data-driven design parameter optimization for the same general class of controller. ADRC was selected because it is model-agnostic and estimates disturbances in real time, a feature well-suited to the patient-to-patient variability that makes vasopressor titration difficult. More broadly, recent work on pressure regulation in ventilator systems has demonstrated that hybrid fuzzy–PID controllers can outperform conventional PID when dealing with uncertain or time-varying plant dynamics [26], reinforcing the value of non-PID architectures for physiological control problems. Finally, the patient-following controller (PFC) was developed as a fully custom design inspired by direct observation of vasopressor response dynamics in the animal data, with the goal of capturing physiological behavior that pre-existing controller frameworks may not accommodate. Together, these five architectures form a deliberate gradient from purely model-free classical control (PID), through hybrid intelligent designs (Step-FIS, AN-FIS, ADRC), to a physiology-inspired custom controller (PFC), and provide a systematic basis for identifying the most suitable approach for automated vasopressor management in hemorrhagic shock.

Two unique PCLC tunings of each controller type were produced using conservative and aggressive tuning schemas, where conservative tuning favored longer rise times to avoid overshooting the target, and aggressive tuning prioritized reduced rise times at the cost of increased overshooting risk. The methods to achieve these performance biases were specific for each controller type and will be covered in their respective subsections. Controller development leveraged vasopressor pressure response dynamic datasets captured in large-animal studies to aid in tuning controller parameters [17]. An initial test scenario for debugging purposes (different to the scenarios that are mentioned in Section 2.4) was used for every controller using the HATRC platform to confirm real-time functionality of each controller.

Aggressive and conservative tuning schemas were prospectively defined based on clinically relevant constraints. Aggressive configurations targeted a rise time of 4–6 min to minimize hypotensive duration, while conservative configurations targeted 7–10 min to reduce overshoot and oscillations [27]. Although these specific rise time targets are engineering design choices, their rationale was grounded in physiological considerations. Prior studies emphasize the dual need to restore arterial pressure promptly to avoid secondary organ injury, while simultaneously avoiding excessive vasoconstriction, masked hypovolemia, and ischemic complications associated with rapid or high-dose vasopressor administration. Clinical guidance commonly targets an MAP near 65 mmHg during damage control resuscitation, and highlights the risks of overly aggressive vasopressor use in hypovolemic states [27].

#### 2.3.1. Proportional–Integral–Derivative Controller

The Proportional–Integral–Derivative (PID)-based controller modulates infusion rates to maintain MAP near the specified target. The control law is derived from a standard PID structure, where the control signal is formed by the weighted sum of the current MAP error, its time integral, and its time derivative. Specifically, the error term is computed as the difference between a delayed estimate of MAP and the target MAP, with the delay modeled as a first-order low-pass filter that captures the physiological latency between drug delivery and hemodynamic response. This filter evolves according to the differential equation, Equation (1):

(1) $$P_{delayed}\left(t+∆t\right)=P_{delayed}+\frac{∆t}{\tau }(P_{measured}-P_{delayed})$$

where τ=15 s, representing the characteristic time constant of the delay.

To improve clinical realism and numerical robustness, the integral term is subject to anti-windup constraints that attenuate accumulation near the setpoint. Specifically, when the absolute MAP error falls below 5 mmHg, the integral accumulator is decayed to reduce excessive gain buildup. The derivative term is computed using a backward difference approximation based on the most recent error value.

In addition, the controller incorporates a rate limiter that bounds the change in infusion rate per time step, ensuring smoother transitions and reducing the risk of abrupt hemodynamic fluctuations. The aggressive PID configuration permits a higher maximum rate of change, favoring faster correction of MAP deviations, and the conservative configuration yields a slower, more gradual response.

Parameter tuning was initially conducted using physiological simulation data from the Pulse Physiology Engine [28] to establish gain stability and responsiveness. Final adjustments and performance verification were performed on the HATRC platform, where the controller was evaluated under a range of hemorrhagic shock conditions to ensure consistent target tracking and minimal overshoot.

#### 2.3.2. Step Fuzzy Inference System

The proposed step fuzzy inference system (Step-FIS) employs a hybrid architecture that integrates a type-2 Sugeno fuzzy logic controller with unit step logic to precisely regulate vasopressor infusion rates. Initially, the measured blood pressure signal is processed through a first-order Butterworth filter to attenuate high-frequency noise and smooth out transient fluctuations. From the resulting filtered signal, the error relative to the target pressure is computed without taking the absolute value; this preserves the sign of the error to clearly indicate whether the current pressure is above or below the target pressure. Concurrently, the derivative of the filtered error is determined to capture the rate at which the error is changing, thereby providing dynamic information on the system’s fluctuating behavior.

These two key parameters—the filtered error and its derivative—form a state space that serves as the input to the fuzzy logic controller. Each input is represented using three membership functions that encode the qualitative behavior of the system. For both error and rate of change, the membership functions consist of:

• Negative—A linear Z-shaped function capturing negative deviations;
• Zero—A triangular function centered around the target condition;
• Positive—A linear S-shaped function representing positive deviations.

**Figure 2:**
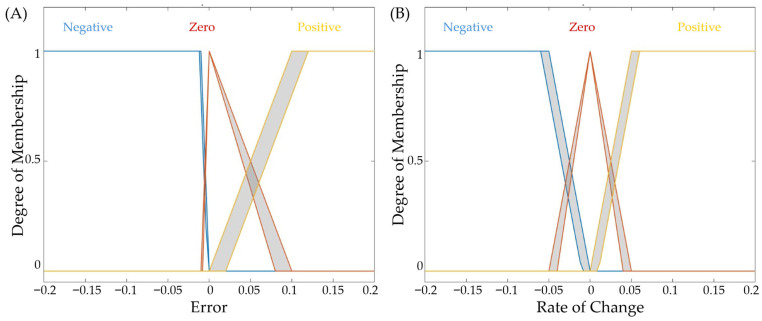
Membership function plots for Step-FIS controller logic.

This structured membership function design, shown in Figure 2, provides smooth transitions in vasopressor rates. Using a type-2 Sugeno framework, the controller effectively addresses uncertainties and variations found in live patient data. A predefined set of IF–THEN rules interprets the state information and generates an output in the range of −1 to 1. This output directs the unit step logic to increase, decrease, or maintain the vasopressor infusion rate, accordingly, ensuring appropriate adjustment in response to both the magnitude and trend of the pressure error.

The complete rule base governing the Step-FIS controller is summarized in Table 2. Five IF–THEN rules map combinations of error and rate-of-change conditions to one of three output actions: increase, maintain, or decrease the vasopressor infusion rate. The rules were designed to enforce a conservative control philosophy: infusion is only increased when the measured pressure is below target and not already rising (Rules 1 and 2). When pressure is actively climbing—regardless of whether the current error is above or below target—the controller holds the current rate to avoid compounding the ongoing response (Rule 3). If the controller detects that the patient has overshot the target (positive error), the infusion rate is decreased with a reduced weight of 0.75 to prevent abrupt withdrawal of vasopressor support (Rule 4). When the error is near zero, the controller maintains the current rate (5). This asymmetric design reflects a clinical preference for gradual dose reduction over aggressive cutbacks, which aligns with guidance on avoiding rebound hypotension during vasopressor weaning.

**Figure 3:**
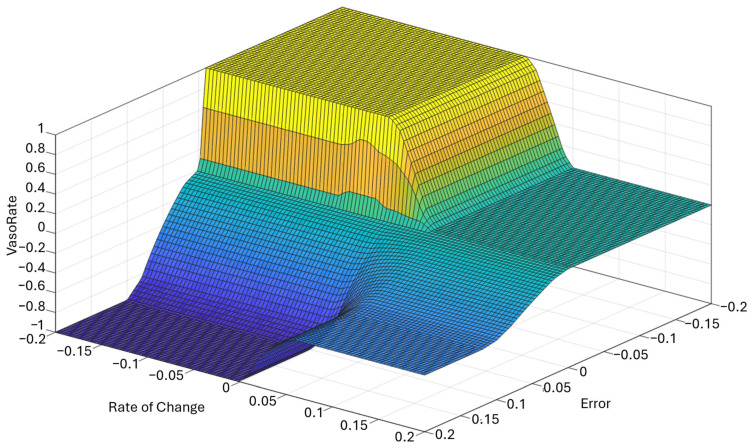
Step-FIS control surface. Color gradient correspondes to the increasing or decreasing of the VasoRate along the vertical axis.

The resulting control surface is shown in Figure 3, which plots the defuzzified output (VasoRate) as a function of both the error and its rate of change. Across the input space, the controller transitions smoothly between increasing, maintaining, and decreasing actions, with the steepest gradients occurring near the boundary between negative and zero error. Both the plateau in the maintain region and the attenuated slope in the decrease region are direct consequences of the rule structure and the reduced weighting of Rule 4, respectively.

Moreover, the control strategy incorporates adaptive step size adjustment to fine-tune the rate adjustments. There are two baseline configurations, aggressive and conservative, with step sizes of 0.035 and 0.025 mL/min, respectively. These initial step sizes were derived from responses observed in the animal data, then used during preliminary controller development to fine-tune the system. To further enhance control performance, the step size was dynamically adjusted: if the filtered error exceeds 30%, the step size is doubled to rapidly counteract significant deviations, whereas if the error is below 3%, the step size is halved to minimize the risk of overshoot. This approach creates a stable response when large corrections are needed while ensuring stability and precision as the pressure converges toward the target. This controller was tested on the HATRC platform to assess performance functionality before fine-tuning and evaluating scenario data.

#### 2.3.3. Adaptive Neural Fuzzy Inference System

Adaptive Neural Fuzzy Inference System (AN-FIS) combines both the rule-based architecture of fuzzy logic (IF–THEN rules) with the learning capabilities of neural networks to adjust the weights of the fuzzy rules based on training data [29]. AN-FIS controller designs have been leveraged successfully for various applications, supporting their possible utility for this effort [30,31,32].

For AN-FIS, the inputs were determined to be the error and rate of change in the error. Given that MAP may vary across subjects, the rate of change can serve as an indicator of how MAP is acting. The output for AN-FIS would be the appropriate vasopressor dosage the system would dictate based on the observed error from target pressure and the rate of change.

AN-FIS selected appropriate vasopressor dosages based on training and tuning performed using animal study data. The full dataset was first aggregated across studies, and outliers were identified and removed to reduce the influence of nonphysiological measurements and improve model robustness. This cleaned dataset was used as the training data for model development. During training, AN-FIS utilized the training data to generate fuzzy rules with generalized bell-shaped membership functions for the input variables and a linear output function [29]. With this, the model was designed as a first-order Sugeno network architecture with 9 rules, and 3 membership functions per variable. Parameter optimization was performed using a hybrid learning algorithm combining least squares estimation for consequent parameters and backpropagation for premise parameters [29]. Training was conducted using the MATLAB R2024b Fuzzy Logic Toolbox, with a maximum number of 100 epochs as a hyperparameter if convergence was not achieved by stabilization of the training error and improvement across successive epochs.

Overfitting was mitigated through multiple strategies, including outlier removal prior to training, careful tuning of the backpropagation learning rate, and study-wise validation using independent animal study datasets not included in the training process. Validation was performed separately using each individual animal study dataset, allowing model generalizability to be assessed across different experimental conditions. Randomization and blinding were not applied during data selection or analysis; instead, all available datasets were used in both training and validation according to the predefined study-wise validation framework. A 70-30 split was applied for training and testing data to evaluate performance on AN-FIS.

To address potential bias in the training data and to ensure physiologically safe controller behavior, additional safeguard mechanisms were incorporated into the dosing logic. Specifically, the AN-FIS output was modulated using a performance-error-based scaling term, such that dosing adjustments were proportionally weighted by the deviation of arterial pressure from the target. A bounded multiplier was applied to constrain the magnitude of dose changes, preventing excessively low or high vasopressor administration while preserving controller responsiveness. These constraints acted as mathematical regularization at the control-output level and were independent of the AN-FIS training process.

This iterative training and validation process was repeated until two AN-FIS systems were selected. The first system produced conservative vasopressor dosages, characterized by minimal dosage adjustments, while the second system produced more aggressive vasopressor dosages to rapidly achieve the target arterial pressure. To further tune and validate the performance, AN-FIS was evaluated on the HATRC platform. AN-FIS was later modified to observe more closely the changes in MAP, more specifically, the rate of change, over time when dosage is given more effectively. Validation of the controller was conducted by using different hemorrhage scenarios and evaluating the Effectiveness and timeliness of the controller at reaching the target pressure.

#### 2.3.4. Active Disturbance Rejection Control

**Figure 4:**
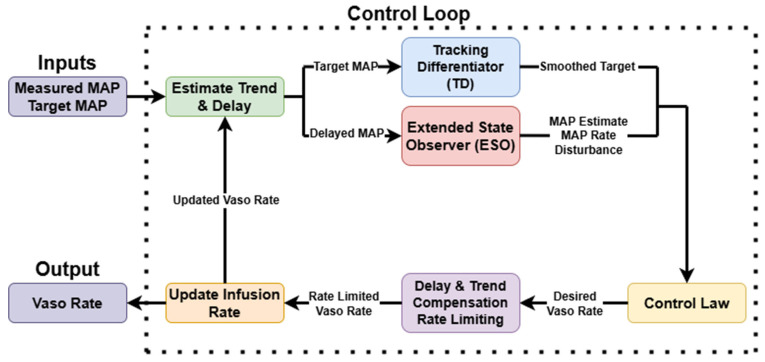
Flow chart of the Active Disturbance Rejection Control logic.

Another vasopressor controller was developed using Active Disturbance Rejection Control (ADRC) [33], a model-agnostic strategy that enables real-time compensation for internal physiological variability and external disturbances. The ADRC architecture comprises three principal components: a tracking differentiator (TD), an extended state observer (ESO), and a state feedback control law (Figure 4).

The TD processes the MAP setpoint into a smoothed reference trajectory and its derivative, facilitating smoother convergence and reducing sensitivity to abrupt setpoint changes. The ESO continuously estimates the true MAP, its rate of change, and a lumped disturbance term that aggregates unmodeled dynamics, patient-specific variability, and external perturbations. These states are updated based on a delayed MAP signal using high-gain observer dynamics, with tuning parameters selected to ensure fast convergence and disturbance rejection.

The control input is computed using a nonlinear feedback law that incorporates both state error and estimated disturbance according to the equation $u\;=\;\frac{\left(-k_{p}\cdot \left(z_{1}-v_{1}\right)-k_{d}\cdot z_{2}+z_{3}\right)}{b}$, where $z_{1}$*,* $z_{2}$, and $z_{3}$ are the ESO state parameters representing the current estimated MAP value, estimated MAP derivative, and estimated disturbance, respectively; $k_{p}$ and $k_{d}$ are the proportional and derivative gains; b is the system gain; and $v_{1}$ is the smoothed reference target. The proportional gain is adapted online as a function of the absolute error magnitude to improve transient performance during large deviations from the target. A delay compensation mechanism modulates the allowable infusion rate adjustment based on the known physiological lag, while trend-aware logic further moderates the rate of change depending on the direction and speed of MAP fluctuations. For instance, when MAP is rapidly rising, the controller restricts further infusion increases to avoid overshooting. Similarly, in cases of steep MAP decline, vasopressor tapering is restrained to preserve perfusion support.

As with the PID controller, ADRC parameters were initially calibrated using simulated data in the Pulse Physiology Engine [28] and refined through empirical testing on the HATRC platform to ensure robustness and fidelity across diverse shock trajectories.

#### 2.3.5. Patient-Following Controller

The custom controller was developed based on characteristics observed in the MAP waveforms following changes in vasopressor dosage captured from animal data. It should be noted that many waveform features were present whether the dosage was increasing or decreasing, with the main difference being a sign change. However, in the present work, the focus was on the ramping (dosage increasing) phase of treatment, so the evaluation will reflect on the vasopressor infusion phase. Additional details are described in Section 4 on future work that will address the weaning (dosage decreasing) phase.

Some key features that were common across all subjects irrespective of dose were (1) most of the MAP change in response to vasopressors occurred within a relatively narrow window, (2) the MAP signals in this region can be approximated as smooth and continuous, and (3) there was a maximum change in MAP effected by any given dose, after which the pressure trended towards steady-state unless and until the system was disturbed again. The real-time MAP responsiveness (e.g., in mmHg/min) was therefore approximated using a five-sample sliding window gradient. Following a dosage change, this value would increase until reaching a local maximum (when MAP was rising the fastest), then decrease until MAP stopped rising due to observation 3 above.

**Figure 5:**
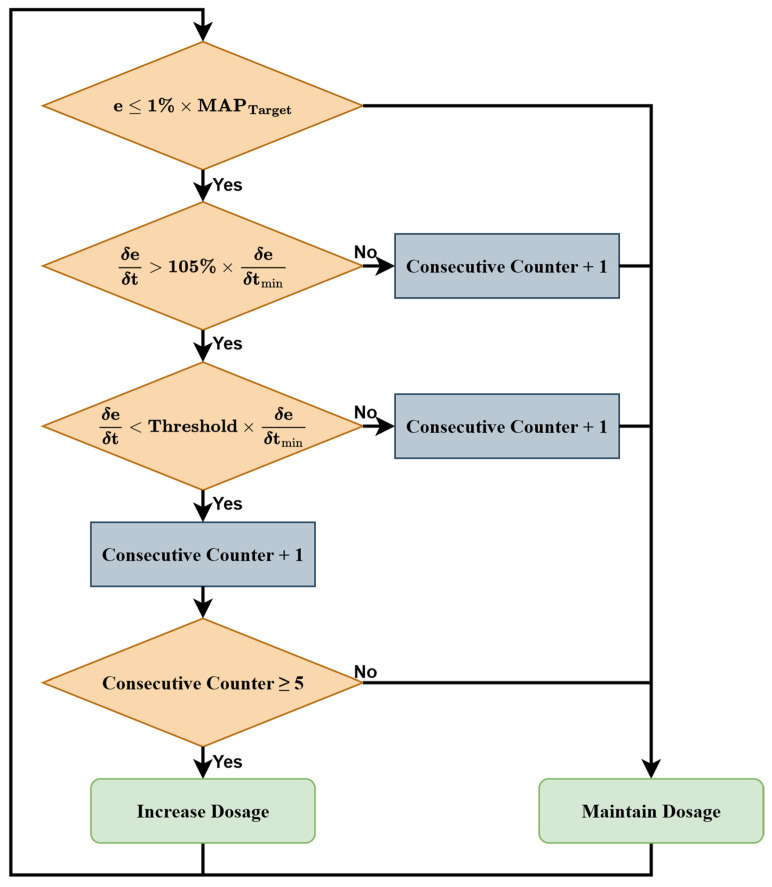
Flow diagram of the patient-following controller logic.

Using this understanding of the system, we built a patient-following controller (PFC) that evaluated the trajectory of the signal error using a conditional rule set to determine the appropriate time to increase the dose (Figure 5). First, a rolling average window was used to smooth the MAP signal, and the smoothed MAP value was converted to a relative error (e) based on the target MAP. When MAP is at the target, this results in an e of 0, and overshoot is indicated by a negative e value. The most recent five samples were used to calculate the error gradient ($\frac{\delta e}{\delta t}$). The smallest $\frac{\delta e}{\delta t}$ value was saved as the current local minimum ($\frac{\delta e}{{\delta t}_{min}}$) and was only replaced by a value that was $\leq 1.05\;*\;\frac{\delta e}{{\delta t}_{min}}$. After the true local minimum, $\frac{\delta e}{\delta t}$ gradually approached 0 as the MAP response slowed, which was used as an indication that the maximum potential effect of the current dose had been reached. If the $\frac{\delta e}{\delta t}$ value crossed a threshold, defined as a certain percentage of $\frac{\delta e}{{\delta t}_{min}}$, for at least 5 consecutive samples, and the MAP was not within 1% of the target, then the dosage was increased by a specified increment. Tuning was accomplished by adjusting the size of the moving average window used for smoothing the MAP (conservative = 10; aggressive = 5), the size of the incremental dosage change (conservative = 0.25 mL/min; aggressive = 0.5 mL/min), and the $\frac{\delta e}{{\delta t}_{min}}$ threshold used for triggering a dosage change (conservative = 0.5; aggressive = 0.9).

### 2.4. Controller Evaluation Using Hardware-in-Loop Test Platform

The test scenarios for comparing PCLC performance used the HATRC platform to simulate a patient requiring vasopressors presenting with a slow internal hemorrhage (10 mL/min), an initial MAP of either 35, 45, or 55 mmHg, and no additional fluid administration during resuscitation. The initial MAP of 35 mmHg was chosen for both the animal data collection and benchtop testing based on previous work by Libert et al., who used it as their hypovolemic pressure for a hemorrhagic-shock model in swine [34,35]. The target MAP of 65 mmHg used for the controllers was based on damage control resuscitation guidelines. To account for controller sensitivities to initiating closer to the target, we subdivided the pressure range in 10 mmHg increments. Each run lasted 30 min, and the hemorrhage was continuous through the entire run. Single test runs were conducted for each controller under each scenario, and the results were analyzed to obtain both standardized and aggregate performance metrics.

### 2.5. Controller Comparison Data Analyses

Data from each scenario run (35, 45, and 55 mmHg starting pressure) for each controller type and configuration were compiled across the entire 30 min run. To qualitatively compare controllers, MAP and vasopressor infusion rates were plotted against time for each PCLC. For a more quantitative evaluation, a series of controller performance metrics were calculated for each controller and scenario. These were used to down-select controller designs similar to methodologies used in other recent studies for closed-loop controllers and machine learning modeling methodologies [22,36,37,38]. These are summarized in Table 3 and have been thoroughly described in the previous literature, except for Maximum Vasopressor Step Change, which was added to quantify the intensity of dosage change each controller used. Each metric was calculated separately across scenarios and controllers, but an aggregate score was calculated by merging metrics into equation terms (Equations (2)–(6)) based on five controller features: (i) stability, (ii) overshoot, (iii) undershoot, (iv) infusion, and (v) EEffectiveness metrics. The overall aggregate score equaled the sum of the stability, overshoot, undershoot, and infusion terms multiplied by the effectiveness term, where the lowest score indicated best performance as shown in Equation (7).

(2) $$“Stable”\;Term=\frac{\left({MDAPE}_{SS}+Wobble+Divergence\right)}{3}$$

(3) $$“Overshoot”\;Term=\frac{\left(TargetOvershoot+AreaAbove\right)}{2}$$

(4) $$“Undershoot”\;Term=\frac{\left(RiseTimeEffeciency+AreaBelow\right)}{2}$$

(5) $$“\mathrm{Infusion}”\;Term=\frac{\left(MeanInfRate+MaxRateChange+VarInfRate\right)}{3}$$

(6) $$“Effectiveness”\;Term=\frac{MDAPE}{Effectiveness}$$

(7) $$Aggregate\;Score=\left(Stable+Overshoot+Undershoot+Infusion\right)*Effectiveness$$

To have each term be dimensionless and weighted similarly to the overall controller performance score, all metrics were normalized to their median score across all PCLCs for each scenario. Median statistics were used instead of mean values to be less sensitive to large outliers due to poor-performing controller designs. Each term shown in Equation (7) was separately calculated for each scenario and averaged across all three scenarios to best quantify the overall controller designs. As the weights for each term were arbitrarily equal, a sensitivity analysis was performed for the aggregate score calculated with Equation (7) to quantify the effects of different parameter weights on overall controller rankings. Each term of the equation was given 2×, 1×, or 0.5× weights to assess how the scores were impacted. Replicates were taken as the performance scores for each scenario (*N* = 3), as each scenario represents a different starting pressure, similar to the variability anticipated between live subjects.

## 3. Results

The results are presented beginning with highlights from each scenario that include plots of MAP and infusion rate vs. time for each PCLC, along with summary tables of performance metrics. Lastly, aggregate scores across all scenarios and controllers are shown to highlight how the controller designs performed overall for down-selecting top controller configurations.

### 3.1. Scenario 1: Starting Pressure 35 mmHg

**Figure 6:**
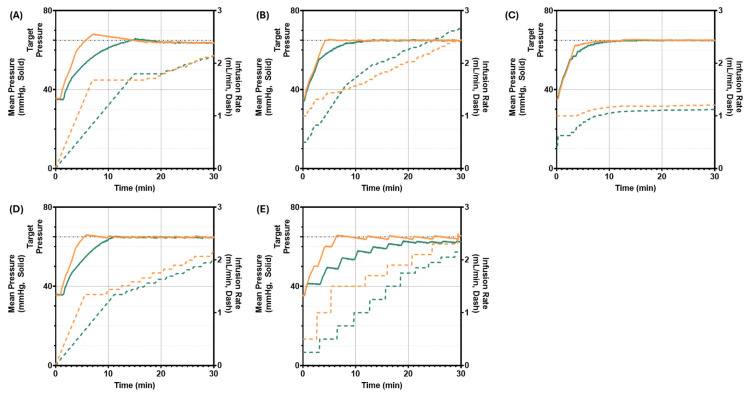
PCLC testing at a starting pressure of 35 mmHg. System pressure (left axis, solid lines) and infusion rate (right axis, dashed lines) are plotted versus scenario run time in minutes. Pressure started at approximately 35 mmHg and each controller had a goal of reaching a 65 mmHg target (black dashed line). More aggressively (orange) and conservatively (green) tuned PCLCs using (**A**) PID, (**B**) Step-FIS, (**C**) AN-FIS, (**D**) ADRC, and (**E**) PFC logic are shown.

Scenario 1 starts at a pressure of 35 mmHg with a continuous slow hemorrhage with a target MAP of 65 mmHg for each controller to reach and maintain for 30 min. Nearly all controllers were able to reach target MAP within the scenario timeframe (Figure 6). However, PID aggressive noticeably overshot target pressure (Figure 6A), with a Target Overshoot percentage of 4.74% (Table 4). PFC conservative undershot the target pressure and did not reach it within the 30 min scenario, the only controller configuration that failed to do so (Figure 6E). Conversely, AN-FIS aggressive reached target pressure the fastest out of all controllers, with a rise time efficiency of 3 min (Figure 6C). Other notable performance differences were MDPE, where each Step-FIS or AN-FIS configuration was more capable of maintaining error close to zero than other controller configurations. More key differences in the PCLC’s performance for scenario 1 are summarized in Table 4.

### 3.2. Scenario 2: Starting Pressure 45 mmHg

**Figure 7:**
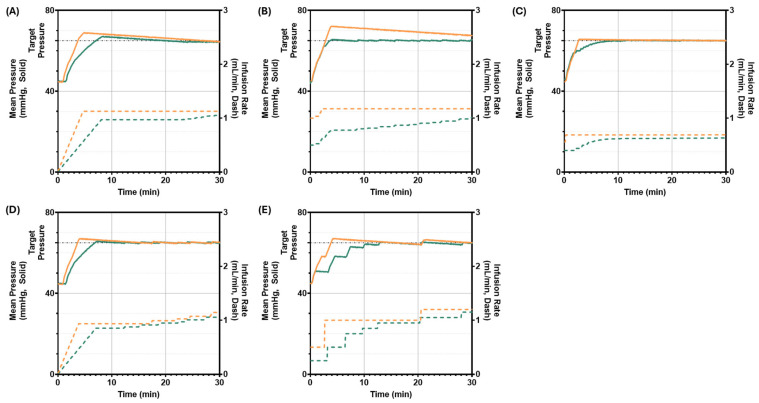
PCLC testing at a starting pressure of 45 mmHg. System pressure (left axis, solid lines) and infusion rate (right axis, dashed lines) are plotted versus scenario run time in minutes. Pressure started at approximately 45 mmHg and each controller had a goal of reaching a 65 mmHg target (black dashed line). More aggressively (orange) and conservatively (green) tuned PCLCs using (**A**) PID, (**B**) Step-FIS, (**C**) AN-FIS, (**D**) ADRC, and (**E**) PFC logic are shown.

The next scenario mirrored the first but with a starting pressure of 45 mmHg, and the closer starting pressure to a target pressure of 65 mmHg led to more PCLC overshooting (Figure 7). For example, it was evident that Step-FIS aggressive strongly overshot the target pressure by a percentage of 11.05% (Figure 7B). No PCLCs undershot the target pressure for the entire scenario duration, but some still took longer to reach the target than others, such as the PFC conservative configuration (Figure 7E). Conservative AN-FIS reached target pressure the fastest, with a rise time efficiency of 1.67 min (Table 5). MDAPE and Effectiveness metrics identified key differences between controller performance, with Step-FIS aggressive having the highest MDAPE at 7.6% and the lowest EEffectiveness at 50.97% (Table 5). Conversely, PID conservative and PFC conservative had the largest area below target pressure. Overall, the AN-FIS aggressive and Step-FIS conservative had the highest Effectiveness, with each surpassing 92% with MDAPE values at or below 0.50%. A summary of all performance metrics for Scenario 2 can be found in Table 4.

### 3.3. Scenario 3: Starting Pressure 55 mmHg

**Figure 8:**
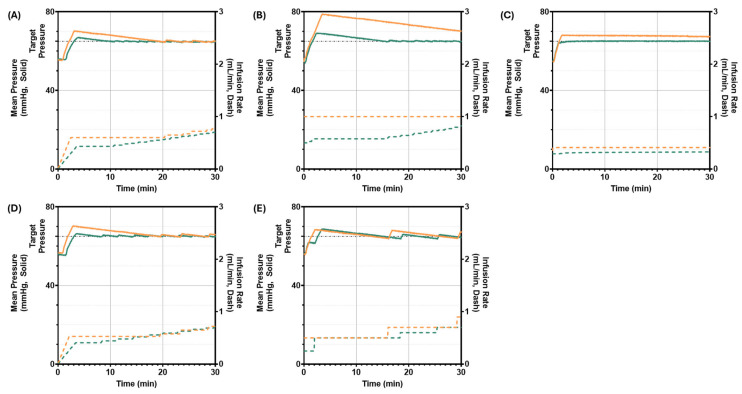
PCLC testing at a starting pressure of 55 mmHg. System pressure (left axis, solid lines) and infusion rate (right axis, dashed lines) are plotted versus scenario run time in minutes. Pressure started at approximately 55 mmHg and each controller had a goal of reaching a 65 mmHg target (black dashed line). More aggressively (orange) and conservatively (green) tuned PCLCs using (**A**) PID, (**B**) Step-FIS, (**C**) AN-FIS, (**D**) ADRC, and (**E**) PFC logic are shown.

The last scenario starts at a pressure of 55 mmHg with a slow hemorrhage and continues for a duration of 30 min. All controllers overshot the target (Figure 8), but Step-FIS aggressive had the largest overshoot of the 65 mmHg target pressure by a percentage of 21.23% (Table 6). Conservative Step-FIS, and both configurations for the PFC, reached the target the fastest with a rise time efficiency of 0.50 min; however, all controllers reached target pressure within one minute, so the differences for this metric were slight (Table 6). Most notable performance differences were evident with MDAPE and the Target Overshoot. Across these two metrics, the AN-FIS conservative controller was most effective at minimizing error with MDAPE at 0.15% with a small Target Overshoot at 0.36%. More details on controller performance metrics for this scenario are summarized in Table 6.

### 3.4. Overall Vasopressor PCLC Performance

We next summarized PCLC performance across all three testing scenarios to identify the most robust controller design. As a number of performance metrics are weighted towards different design features, we developed an aggregate controller metric which groups a wide range of performance metrics related to “stability”, “overshoot”, “undershoot”, and “infusion” equally, relative to an “effectiveness” term (see Equations (2)–(7)). In doing so, each of these terms could be separately evaluated as well as the aggregate score, where the lowest score is the best performance.

**Figure 9:**
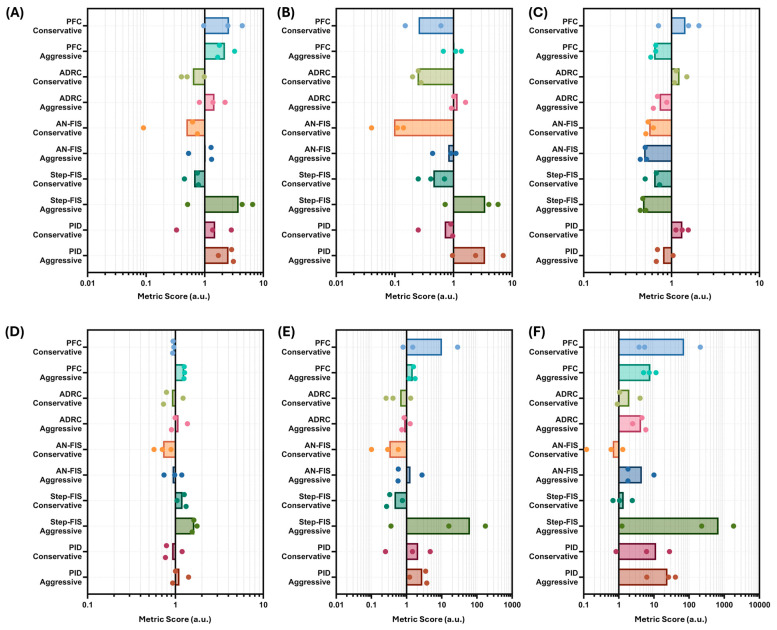
Comparison of average PCLC performance. Compiled terms for (**A**) stable, (**B**) overshoot, (**C**) undershoot, (**D**) infusion, and (**E**) Effectiveness terms separately, and (**F**) overall performance scores. Results were averaged across three scenario runs, with individual replicates for each scenario shown. Each plot’s x-axis is logarithmic due to the wide range of scores across each controller.

For the “Stable” term, the Step-FIS aggressive had the worst performance, followed by the PFC conservative configuration; however, each was highly variable across the three trial runs (Figure 9A). Conversely, the top three performing controllers for this term were AN-FIS conservative, Step-FIS conservative, and ADRC conservative, highlighting the preference for the more conservative controller types. For the “Overshoot” term, the AN-FIS controller performed the best and was significantly lower than the second-best controller, the ADRC conservative configuration (Figure 9B). This once again highlights the metric preference for conservatively tuned controllers, which was logical, as this term was based around avoiding overshoot. Step-FIS and PID aggressive controller configurations had the worst performance for this term. The “Undershoot” term was developed to prioritize performance, where less time is spent below target MAP. As such, the AN-FIS and Step-FIS aggressive configurations performed the best, while AN-FIS conservative still performed well, ranking in the top three for this term (Figure 9C). The “infusion” term summarized metrics related to vasopressor infusion, max rate of change, mean infusion rate, and variable infusion rate. For this term, AN-FIS, PID, and ADRC conservative configurations performed best while all aggressive configurations performed worse than their conservative configuration counterparts (Figure 9D). Each term is proportional to the “effectiveness” term, where large differences were evident for controller configurations (Figure 9E). AN-FIS, Step-FIS, and ADRC conservative configurations performed the best, while Step-FIS aggressive and PID conservative had much larger values for this term.

Summarizing the overall performance using the aggregate metric, three PCLC configurations had scores below 2—AN-FIS conservative (0.67), Step-FIS conservative (1.38), and ADRC conservative (1.99)—with the next closest controller scores being more than two times higher (ADRC aggressive [4.29] and AN-FIS aggressive [4.53]) (Figure 9F). Each of these metrics is compiled in Table 7 for each scenario and across the entire study for each controller type and configuration.

**Figure 10:**
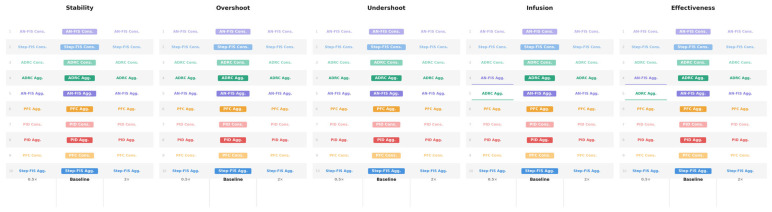
Aggregate score sensitivity analysis. Overall aggregate score ranking across the tested controllers when each term’s weight is independently adjusted to be 2× or 0.5× of its original weight. Results are shown (from left to right) for the “Stability”, “Overshoot”, “Undershoot”, “Infusion”, and “Effectiveness” terms. Ranks are shown from best to worst performing controllers for 0.5×, Baseline or 1×, and 2× weight adjustments for each. Underlined controller names indicate a rank adjustment when the weight was adjusted.

Aggregate score sensitivity analysis evaluated the effects of adjusting weights of each formula sub-component on overall controller ranking results (Figure 10). For the “Stability”, “Overshoot”, or “Undershoot” terms, increasing or decreasing the term importance by 2× or 0.5×, respectively, had no impact on controller performance ranking. The “Infusion” and “Effectiveness” terms had one ranking adjustment when their weights were decreased, with the AN-FIS Agg. and ADRC Agg. controllers changing overall rankings, which were the fourth- and fifth-strongest-performing controllers. Doubling these terms’ weights did not adjust the overall comparison ranks for testing controllers. Overall, the aggregate score had low sensitivity to adjusting how the score was calculated, highlighting the robustness of the top-performing controllers.

## 4. Discussion

Vasopressor therapy can be a critical adjuvant to infusion therapy during hemorrhage management. However, it can be challenging to effectively manage during intensive care hospital settings, let alone trauma situations in the pre-hospital civilian or military environment. As such, PCLCs for effectively managing treatment can simplify this life-saving therapy if they can be shown to perform robustly across a wide range of hemorrhage scenarios. This research effort takes the first steps toward the design of vasopressor PCLCs suitable for hemorrhage care, comparing a wide range of controller configurations using a hardware-in-loop test platform.

The evaluation of our controllers tended to favor conservatively tuned systems. In our study, the AN-FIS controller achieved the top overall performance with an aggregate score of 0.67, followed by the STEP-FIS at 1.38 and the ADRC at 1.99. These results indicate that conservative tuning may yield a preferable response for fluid non-responsiveness. Aggressively tuned variants generally underperformed their conservative counterparts, especially when the starting pressure was close to target pressure, suggesting that the inherent risks of overshooting and instability become prominent in these situations.

In clinical contexts, overshoot is associated with risks, such as exacerbation of ischemic injuries. While our simulation cannot replicate these fully, minimizing overshoot remains a priority [44], since high pressures may exacerbate hemorrhage by disrupting clots [45]. Target undershoot is important to avoid during hemorrhagic shock, as vasopressor under-infusion will leave the casualty hypotensive, risking progression to irreversible shock [46]. Regarding stability, vasopressor controllers should aim to have high stability, as low stability manifests as oscillations in blood pressure. Such oscillations can cause rebleeding by generating shear stress on the vasculature, potentially dislodging clots forming at injury sites [47]. Regarding infusion, controllers must balance the speed of vasopressor delivery against the total dose to avoid excessive vasoconstriction [27]. While excessive vasoconstriction may produce an acceptable MAP on the patient monitor, cardiac output may decrease as the heart is forced to pump against elevated systemic vascular resistance, imposing additional cardiac strain in an already compromised hypovolemic state [44]. Finally, controller Effectiveness maintains MAP within a therapeutic window that supports organ perfusion without excessive vasopressor dosing. Poor controller Effectiveness, characterized by prolonged periods outside this safe range, risks progression to irreversible shock [48]. These potential complications also highlight the sensitive balance that must be held between patient state, target setting, and resource availability.

The optimal weighting of each performance area is an open area for future research that will refine controller development. However, sensitivity analysis results indicated that doubling or halving the weight of each term had minimal impact on controller ranking, indicating that the results of this study and the use of the aggregate score are robust to these concerns. It is worth noting that without better informed scoring, the controller tuning for the aggressive and conservative configurations is subjective and cannot be directly correlated to more favorable patient outcomes. The control architectures considered here, including the best performing ones, should also not be assumed to be the ideal models for this application. While they each have benefits that earned them a place in this study, there may yet be more-optimal models that will perform better across more diverse patients and injury states. Given the large impact on controller performance, future explorations should consider incorporating a broader range of tuning levels to balance responsiveness with system stability.

The analysis of scenario-specific performance further elucidates the nuances in controller behavior. In the first scenario, characterized by an initial pressure of 35 mmHg, the aggressively tuned Step-FIS controller demonstrated superior immediate performance, aligning closely with the optimal initial-rate condition. However, its success in the early phase translated into poor overall performance, as the aggressive setting led to significant overshoot in subsequent conditions. This observation highlights that an optimal initial rate does not necessarily confer lasting benefits; instead, it underscores the need for a dynamic tuning strategy. Incorporating adaptive initial-rate selection could allow for adjustments in real time, thereby mitigating overshoot and improving the controller’s performance across a broader range of situations. Another consideration is the impact that anesthesia depth and agent may have on vasopressor responsiveness, since some anesthetic agents alter vascular tone and thereby change the patient’s dosage sensitivity. Data on this matter could be implemented in future versions of V-ARC to make the system more adaptive. Furthermore, extending the testing conditions to encompass variable hemorrhage rates, differing vasopressor Effectiveness, and the simultaneous use of traditional infusates alongside vasopressor therapy would likely provide a more holistic performance profile. An additional limitation is that the controllers were developed treating NE dosage as equivalent to the controllers’ output of infusion rate. This is not problematic if the same concentration of vasopressor solution will be used as that of the training datasets. However, this may not always be the case, so changing to a more generalized unit for medication dosing would expand the functionality and simplify deployment for the end-user.

The utility of the test platform used in this research also deserves critical reflection. The platform offers multiple testing approaches, ranging from variations in hemorrhage rates to the intentional introduction of noise, to challenge the robustness of each controller. Despite these strengths, a key limitation remains: the current system only simulates the effects of norepinephrine in pigs on a pressure response level. The VCM only induces pressure changes by choking the circulatory flow and does not replicate the tachycardiac effects of NE or the changes in cardiac output. This simplification limits our ability to predict real-world performance where patient-specific, complex physiological responses can significantly impact outcomes, particularly in multi-trauma casualties that may be mechanically ventilated, as cardiac preload and ventilation pressures must be delicately balanced. To bridge this gap, future work will integrate animal testing and further refine the platform to mimic broader clinical conditions and expand the physiological fidelity of the model. An example of one such improvement that is being investigated, but falls outside the scope of the present work, is the combination of fluid therapy with vasopressor administration. Increasing circulating volume is still the most effective means of treating hemorrhagic shock, so deploying vasopressors for supplemental, rather than primary, support should be thoroughly examined. Such enhancements would not only validate the current findings but also drive the design of control strategies that are robust and translatable to human applications.

## 5. Conclusions

Hemorrhagic shock remains a leading cause of death during trauma and can be complicated by fluid non-responsiveness or resource-constrained situations where traditional fluid therapy alone may be insufficient to stabilize a casualty. PCLCs for vasopressor administration may be able to aid in these situations if they can be tuned for effective vasopressor administration. Overall, our results clearly demonstrate that more conservatively tuned controller designs provide a more reliable framework for strong controller performance under varied conditions. The performance degradation observed with aggressive tuning, despite occasional scenario-specific advantages, suggests that adaptive- and dynamic-tuning strategies should be the focus of subsequent research. By broadening the range of scenarios and incorporating more-realistic test conditions, future studies can build on these findings to develop PCLCs that are not only robust but also finely tuned to the intricacies of clinical use. Ultimately, these efforts demonstrate that conservative controller designs offer improved performance in simulated hemorrhage scenarios, providing a foundation for future in vivo evaluation of PCLCs for vasopressor administration.

## Figures and Tables

**Table 1:** Summary of norepinephrine dosage used during hemorrhage resuscitation in swine.

Step	Norepinephrine Dose (mcg/min)	Norepinephrine Rate (mL/min)
1	0.5	0.13
2	1	0.25
3	2	0.5
4	4	1
5	8	2
6	12	3
7	16	4
8	20	5

**Table 2:** Step-FIS rule base.

Rule	Error	Rate of Change	Output (VasoRate)	Weight
1	Negative	Zero	Increase	1.00
2	Negative	Negative	Increase	1.00
3	Any	Positive	Maintain	1.00
4	Positive	Any	Decrease	0.75
5	Zero	Any	Maintain	1.00

**Table 3:** Summary of performance metrics.

Performance Metrics	Description	Reference
Median Performance Error (MDPE, %)	Median value of the performance errors vs. target pressure	[38,39]
Median Absolute Performance Error (MDAPE, %)	Median of the absolute value of performance errors vs. target pressure	[39]
MDAPE at Steady-State (MDAPE_SS_, %)	MDAPE values once the controller has reached steady-state	[40]
Target Overshoot (%)	Percent by which the target was maximally overshot	[41]
Effectiveness (%)	Percentage of the time the controller was within +/−5 mmHg of the target	[42]
Wobble (%)	Measurement of the dispersion of performance errors, relative to MDPE	[38,39]
Divergence (%/h)	Measurement of the trend of MDAPE over the final 10% of the scenario	[38]
Rise Time Efficiency (min)	Time for the controller to initially be within 90% of target MAP	[41]
Mean Infusion Rate (MeanInf, mL/min)	Average of the infusion rates used during the scenario	[22]
Area Above Target Pressure (AreaAbove, min)	Quantifies over-resuscitation using cumulative area of the time spent above target MAP during scenario	[22]
Area Below Target Pressure (AreaBelow, min)	Quantifies under-resuscitation using cumulative area of the time spent below target MAP (after reaching target for the first time) during scenario	[22]
Maximum Infusion Rate Change (MaxRateChange, mL/min/min)	Quantifies the maximum time of change in the infusion rate across the test using a 3 min moving-average window	N/A
Variable Infusion Rate (VarInfRate, mL/min)	Standard deviation of a 2 min window of infusion rates relative to mean infusion rate	[43]

**Table 4:** Summary of PCLC performance for starting MAP of 35 mmHg.

		PID		Step-FIS		AN-FIS		ADRC		PFC	
		Agg.	Cons.	Agg.	Cons.	Agg.	Cons.	Agg.	Cons.	Agg.	Cons.
Performance Metrics	MDPE (%)	−1.53	−2.07	−0.12	−0.38	−0.13	−0.31	−0.29	−0.61	−0.85	−8.36
	MDAPE (%)	1.90	2.07	0.20	0.39	0.33	0.31	0.47	0.61	0.94	8.36
	MDAPE_SS_ (%)	1.77	1.73	0.17	0.25	0.27	0.21	0.35	0.46	0.70	4.31
	Target Overshoot (%)	4.74	1.26	0.65	0.44	0.60	0.21	1.53	0.39	1.23	0.00
	Effectiveness (%)	86.35	69.64	88.58	81.62	89.42	84.95	86.64	74.10	85.23	46.79
	Wobble (%)	0.87	0.43	0.17	0.25	0.27	0.20	0.32	0.21	0.66	0.48
	Divergence (%/h)	3.86	5.69	−1.12	1.87	−0.04	1.36	9.76	−2.27	11.79	−1.60
	Rise Time Efficiency (min)	3.75	8.10	3.25	4.51	3.00	3.92	3.76	7.10	3.92	13.10
	MeanInf (ml/min)	1.59	1.41	1.80	1.87	1.15	1.01	1.51	1.28	1.68	1.26
	AreaAbove (min)	0.22	0.01	0.02	0.01	0.03	0.00	0.04	0.00	0.03	0.00
	AreaBelow (min)	−1.50	−2.69	−1.00	−1.47	−1.05	−1.24	−1.30	−2.23	−1.38	−4.01
	MaxRateChange (mL/min^2^)	0.0041	0.0020	0.0182	0.0094	0.0167	0.01	0.0041	0.0020	0.0083	0.0042
	VarInfRate (mL/min)	0.0448	0.0432	0.0299	0.0427	0.0040	0.0200	0.0431	0.0422	0.0546	0.0542

**Table 5:** Summary of PCLC performance for starting MAP of 45 mmHg.

		PID		Step-FIS		AN-FIS		ADRC		PFC	
		Agg.	Cons.	Agg.	Cons.	Agg.	Cons.	Agg.	Cons.	Agg.	Cons.
Performance Metrics	MDPE (%)	2.25	−0.53	7.02	0.07	0.42	−0.03	0.28	−0.04	0.79	−1.08
	MDAPE (%)	3.16	1.16	7.60	0.23	0.50	0.25	0.62	0.32	1.32	1.08
	MDAPE_SS_ (%)	2.63	0.94	7.42	0.20	0.46	0.21	0.47	0.24	1.13	0.63
	Target Overshoot (%)	6.15	3.21	11.05	0.97	1.19	0.51	3.14	1.19	3.24	0.75
	Effectiveness (%)	89.97	83.84	50.97	92.75	93.32	91.36	89.97	83.84	89.69	76.60
	Wobble (%)	1.72	1.11	1.78	0.20	0.23	0.20	0.50	0.24	0.86	0.58
	Divergence (%/h)	13.30	2.58	−17.15	2.62	−1.01	−0.17	7.09	−5.12	−13.47	−31.31
	Rise Time Efficiency (min)	2.75	4.34	2.00	2.00	1.84	1.67	2.75	4.26	2.92	6.76
	MeanInf (ml/min)	1.04	0.85	1.17	0.83	0.69	0.59	0.92	0.82	1.02	0.83
	AreaAbove (min)	0.71	0.20	2.00	0.05	0.13	0.03	0.21	0.05	0.30	0.01
	AreaBelow (min)	−0.70	−1.17	−0.45	−0.50	−0.43	−0.60	−0.70	−1.10	−0.60	−1.46
	MaxRateChange (mL/min^2^)	0.0041	0.0020	0.0171	0.0086	0.0108	0.01	0.0041	0.0020	0.0083	0.0042
	VarInfRate (mL/min)	0.0219	0.0208	0.0028	0.0115	0.0030	0.0044	0.0237	0.0222	0.0219	0.0238

**Table 6:** Summary of PCLC performance for starting MAP of 55 mmHg.

		PID		Step-FIS		AN-FIS		ADRC		PFC	
		Agg.	Cons.	Agg.	Cons.	Agg.	Cons.	Agg.	Cons.	Agg.	Cons.
Performance Metrics	MDPE (%)	1.04	−0.18	14.13	0.33	4.33	0.09	1.59	0.21	1.58	0.55
	MDAPE (%)	1.84	0.38	14.18	0.53	4.35	0.15	1.90	0.40	1.86	1.26
	MDAPE_SS_ (%)	1.38	0.33	11.00	0.40	4.34	0.15	1.71	0.37	1.78	1.16
	Target Overshoot (%)	8.04	3.00	21.23	6.34	4.80	0.36	8.14	2.08	5.23	5.76
	Effectiveness (%)	92.49	93.32	5.01	96.94	97.49	97.77	92.48	93.05	97.49	97.78
	Wobble (%)	1.77	0.29	1.48	0.56	0.23	0.13	1.54	0.35	1.63	1.33
	Divergence (%/h)	0.76	3.46	−28.51	0.89	−2.73	0.06	35.47	2.18	34.16	1.92
	Rise Time Efficiency (min)	1.17	1.75	0.50	0.75	0.58	0.58	1.09	1.75	0.50	0.50
	MeanInf (ml/min)	0.61	0.50	1.00	0.63	0.41	0.32	0.55	0.50	0.60	0.54
	AreaAbove (min)	0.71	0.10	4.09	0.47	1.22	0.03	0.77	0.11	0.54	0.39
	AreaBelow (min)	−0.24	−0.36	−0.10	−0.16	−0.12	−0.13	−0.18	−0.33	−0.17	−0.24
	MaxRateChange (mL/min^2^)	0.0041	0.0020	0.0167	0.0086	0.0065	0.00	0.0041	0.0020	0.0083	0.0048
	VarInfRate (mL/min)	0.0155	0.0154	0.0000	0.0070	0.0008	0.0008	0.0155	0.0160	0.0067	0.0057

**Table 7:** Summary of aggregate performance metrics for all PCLC configurations across each scenario (35, 45, or 55 mmHg) and the overall study.

		PID		Step-FIS		AN-FIS		ADRC		PFC	
		Agg.	Cons.	Agg.	Cons.	Agg.	Cons.	Agg.	Cons.	Agg.	Cons.
35 mmhg	Stable Term	3.08	2.84	0.51	0.78	0.53	0.62	2.22	0.98	3.23	4.37
	Overshoot Term	7.02	0.97	0.72	0.41	0.90	0.11	1.60	0.25	1.36	0.00
	Undershoot Term	0.67	1.32	0.51	0.73	0.50	0.62	0.62	1.13	0.66	2.05
	Infusion Term	0.93	0.77	1.62	1.26	1.18	0.89	0.90	0.73	1.25	0.93
	Effectiveness Term	3.47	4.68	0.36	0.76	0.58	0.58	0.85	1.30	1.75	28.21
	Overall Score	40.61	27.60	1.23	2.41	1.81	1.29	4.56	4.01	11.36	207.45
45 mmhg	Stable Term	2.87	1.36	6.56	0.75	1.31	0.75	0.81	0.40	1.66	0.96
	Overshoot Term	2.38	0.90	5.74	0.25	0.44	0.14	0.92	0.28	1.09	0.15
	Undershoot Term	0.69	1.12	0.47	0.50	0.44	0.51	0.69	1.08	0.66	1.56
	Infusion Term	1.00	0.79	1.55	1.04	0.98	0.71	0.99	0.79	1.26	0.96
	Effectiveness Term	3.73	1.47	15.85	0.27	0.57	0.29	0.73	0.41	1.57	1.49
	Overall Score	25.90	6.13	227.08	0.68	1.82	0.60	2.47	1.05	7.33	5.41
55 mmhg	Stable Term	1.71	0.33	4.35	0.45	1.28	0.09	1.38	0.50	1.78	2.48
	Overshoot Term	0.96	0.25	4.00	0.70	1.10	0.04	1.01	0.20	0.67	0.61
	Undershoot Term	1.04	1.55	0.44	0.67	0.52	0.54	0.88	1.49	0.58	0.71
	Infusion Term	1.41	1.19	1.76	1.32	0.74	0.57	1.37	1.22	1.27	0.94
	Effectiveness Term	1.22	0.25	173.65	0.33	2.74	0.10	1.26	0.26	1.17	0.79
	Overall Score	6.24	0.83	1833.27	1.05	9.96	0.12	5.84	0.90	5.04	3.75
Average	Stable Term	2.55	1.51	3.81	0.66	1.04	0.49	1.47	0.63	2.22	2.60
	Overshoot Term	3.45	0.71	3.49	0.45	0.81	0.10	1.17	0.24	1.04	0.25
	Undershoot Term	0.80	1.33	0.47	0.63	0.49	0.56	0.73	1.23	0.63	1.44
	Infusion Term	1.11	0.92	1.64	1.21	0.97	0.72	1.08	0.92	1.26	0.94
	Effectiveness Term	2.81	2.13	63.29	0.45	1.30	0.32	0.95	0.66	1.50	10.16
	Overall Score	24.25	11.52	687.19	1.38	4.53	0.67	4.29	1.99	7.91	72.21

## Data Availability

The data presented in this study and tuned controller parameters are not publicly available because they have been collected and maintained in a government-controlled database located at the U.S. Army Institute of Surgical Research. This data can be made available through the development of a Cooperative Research and Development Agreement (CRADA) with the corresponding author.

## Abbreviations

The following abbreviations are used in this manuscript:

AAALAC	Association for Assessment and Accreditation of Laboratory Animal Care International
AN-FIS	Adaptive Neural Inference System
ADRC	Active Disturbance Rejection Control
ANOVA	Analysis of Variance
ARC	Adaptive Resuscitation Controller
AreaAbove	Area Above Target Pressure
AreaBelow	Area Below Target Pressure
ESO	Extended State Observer
HATRC	Hardware-in-Loop Testbed for Resuscitation Controllers
IACUC	Institutional Animal Care and Use Committee
IMC	Internal Model Controller
LPV	Linear Parameter-Varying
MAP	Mean Arterial Pressure
MaxRateChange	Maximum Infusion Rate Change
MDAPE	Median Absolute Performance Error
MDAPESS	Median Absolute Performance at Steady-State
MDPE	Median Performance Error
MeanInf	Mean Vasopressor Infusion Rate
MMEKF	Multiple-Model Extended Kalman Filter
NE	Norepinephrine
PCLC	Physiological Closed-Loop Controller
PFC	Patient-Following Controller

## References

[1] Baykuziyev T., Khan M.J., Karmakar A., Baloch M.A. (2023). Closed-loop pharmacologic control of blood pressure: A review of existing systems. Cureus.

[2] Brogi E., Cyr S., Kazan R., Giunta F., Hemmerling T.M. (2017). Clinical performance and safety of closed-loop systems: A systematic review and meta-analysis of randomized controlled trials. Anesth. Analg..

[3] Schaffer C., Goldart E., Ligsay A., Mazwi M., Gallant S., Ehrmann D. (2023). Take a load off: Understanding, measuring, and reducing cognitive load for cardiologists in high-stakes care environments. Curr. Treat. Options Pediatr..

[4] von Platen P., Pomprapa A., Lachmann B., Leonhardt S. (2020). The dawn of physiological closed-loop ventilation—A review. Crit. Care.

[5] Pasha S., Babar E.T.R., Schneider J., Heithaus J., Mujeeb-U-Rahman M. A Low-cost, Automated, Portable Mechanical Ventilator for Developing World. Proceedings of the 2021 IEEE Global Humanitarian Technology Conference (GHTC).

[6] Snider E.J., Vega S.J., Nessen I.A., Hernandez Torres S., Salazar S., Berard D., Salinas J. (2024). In Vivo Evaluation of an Adaptive Resuscitation Controller Using Whole Blood and Crystalloid Infusates for Hemorrhagic Shock. Front. Bioeng. Biotechnol..

[7] Vega S.J., Berard D., Avital G., Ross E., Snider E.J. (2023). Adaptive closed-loop resuscitation controllers for hemorrhagic shock resuscitation. Transfusion.

[8] Russell J.A. (2019). Vasopressor therapy in critically ill patients with shock. Intensive Care Med..

[9] Bauer S.R., Gellatly R.M., Erstad B.L. (2023). Precision fluid and vasoactive drug therapy for critically ill patients. Pharmacother. J. Hum. Pharmacol. Drug Ther..

[10] De Backer D., Biston P., Devriendt J., Madl C., Chochrad D., Aldecoa C., Brasseur A., Defrance P., Gottignies P., Vincent J.-L. (2010). Comparison of dopamine and norepinephrine in the treatment of shock. N. Engl. J. Med..

[11] Sviri S., Hashoul J., Stav I., Van Heerden P.V. (2014). Does high-dose vasopressor therapy in medical intensive care patients indicate what we already suspect?. J. Crit. Care.

[12] Padmanaban A., Venkataraman R., Rajagopal S., Devaprasad D., Ramakrishnan N. (2020). Feasibility and safety of peripheral intravenous administration of vasopressor agents in resource-limited settings. J. Crit. Care Med..

[13] Rinehart J., Ma M., Calderon M.-D., Cannesson M. (2018). Feasibility of automated titration of vasopressor infusions using a novel closed-loop controller. J. Clin. Monit. Comput..

[14] Wassar T., Lupsay T.G., Kallu U.R., Moisi M., Voigt R.B., Marques N.R., Khan M.N., Grigoriadis K.M., Kramer G.C., Franchek M.A. (2013). Automatic Control of Mean Arterial Pressure During Trauma Resuscitation Using Closed-Loop Vasopressor Therapy. System.

[15] Luspay T., Grigoriadis K. (2015). Robust linear parameter-varying control of blood pressure using vasoactive drugs. Int. J. Control.

[16] Coeckelenbergh S., Soucy-Proulx M., Van der Linden P., Clanet M., Rinehart J., Cannesson M., Duranteau J., Joosten A. (2024). Tight control of mean arterial pressure using a closed loop system for norepinephrine infusion after high-risk abdominal surgery: A randomized controlled trial. J. Clin. Monit. Comput..

[17] Joosten A., Rinehart J., Cannesson M., Coeckelenbergh S., Pochard J., Vicaut E., Duranteau J. (2024). Control of mean arterial pressure using a closed-loop system for norepinephrine infusion in severe brain injury patients: The COMAT randomized controlled trial. J. Clin. Monit. Comput..

[18] Merouani M., Guignard B., Vincent F., Borron S.W., Karoubi P., Fosse J.-P., Cohen Y., Clec’h C., Vicaut E., Marbeuf-Gueye C. (2008). Norepinephrine weaning in septic shock patients by closed loop control based on fuzzy logic. Crit. Care.

[19] Pandey S., Singh A.K., Kulshreshtha S.B. (2025). Design of an Intelligent Fuzzy Controller for Drug Dosage Optimization in ICU Patients. Int. Adv. Res. J. Sci. Eng. Technol..

[20] Zhao Y.F., Chaw J.K., Ang M.C., Tew Y., Shi X.Y., Liu L., Cheng X. (2025). A safe-enhanced fully closed-loop artificial pancreas controller based on deep reinforcement learning. PLoS ONE.

[21] Berard D., Lopez M.D., Ruiz A., Bermudez J.M., Gathright R., Rodgers T.M., Torres S.I.H., Shimoura C.G., Ross E., Snider E.J. (2025). Vasopressor Control Module Development and Integration into the Hardware-in-Loop Automated Testbed for Resuscitation Controller Evaluation. Res. Sq..

[22] Snider E.J., Berard D., Vega S.J., Hernandez Torres S.I., Avital G., Boice E.N. (2022). An Automated Hardware-in-Loop Testbed for Evaluating Hemorrhagic Shock Resuscitation Controllers. Bioengineering.

[23] Berard D., Lopez M.D., Ruiz A., Marrero Bermudez J., Gathright R., Rodgers T.M., Hernandez Torres S.I., Shimoura C.G., Ross E., Snider E.J. (2026). Development of a vasopressor control module for testing hemorrhagic shock resuscitation controllers. Biomed. Eng. OnLine.

[24] Rodgers T.M., Berard D., Gonzalez J.M., Vega S.J., Gathright R., Bedolla C., Ross E., Snider E.J. (2024). In Vivo Evaluation of Two Hemorrhagic Shock Resuscitation Controllers with Non-Invasive, Intermittent Sensors. Bioengineering.

[25] Huang J.W., Roy R.J. (1998). Multiple-drug hemodynamic control using fuzzy decision theory. IEEE Trans. Biomed. Eng..

[26] Acharya D., Das D.K. (2023). A novel PID controller for pressure control of artificial ventilator using optimal rule based fuzzy inference system with RCTO algorithm. Sci. Rep..

[27] Beloncle F., Meziani F., Lerolle N., Radermacher P., Asfar P. (2013). Does vasopressor therapy have an indication in hemorrhagic shock?. Ann. Intensive Care.

[28] Bray A., Webb J.B., Enquobahrie A., Vicory J., Heneghan J., Hubal R., TerMaath S., Asare P., Clipp R.B. (2019). Pulse Physiology Engine: An Open-Source Software Platform for Computational Modeling of Human Medical Simulation. SN Compr. Clin. Med..

[29] Jang J.-S. (1993). ANFIS: Adaptive-network-based fuzzy inference system. IEEE Trans. Syst. Man Cybern..

[30] Basar G., Der O., Guvenc M.A. (2025). AI-powered hybrid metaheuristic optimization for predicting surface roughness and kerf width in CO_2_ laser cutting of 3D-printed PLA-CF composites. J. Thermoplast. Compos. Mater..

[31] Islam M.A., Singh J.G., Jahan I., Lipu M.H., Jamal T., Elavarasan R.M., Mihet-Popa L. (2021). Modeling and performance evaluation of ANFIS controller-based bidirectional power management scheme in plug-in electric vehicles integrated with electric grid. IEEE Access.

[32] Jumani T.A., Mustafa M.W., Hamadneh N.N., Atawneh S.H., Rasid M.M., Mirjat N.H., Bhayo M.A., Khan I., Jumani T.A., Mustafa M.W. (2020). Computational Intelligence-Based Optimization Methods for Power Quality and Dynamic Response Enhancement of ac Microgrids. Energies.

[33] Han J. (2009). From PID to active disturbance rejection control. IEEE Trans. Ind. Electron..

[34] Libert N., Chenegros G., Harrois A., Baudry N., Cordurie G., Benosman R., Vicaut E., Duranteau J. (2018). Performance of closed-loop resuscitation of haemorrhagic shock with fluid alone or in combination with norepinephrine: An experimental study. Ann. Intensive Care.

[35] Libert N., Chenegros G., Harrois A., Baudry N., Decante B., Cordurie G., Benosman R., Mercier O., Vicaut E., Duranteau J. (2021). Performance of closed-loop resuscitation in a pig model of haemorrhagic shock with fluid alone or in combination with norepinephrine, a pilot study. J. Clin. Monit. Comput..

[36] Der O., Tasci M., Basar G., Ercetin A. (2025). Intelligent modeling and prediction of CO2 laser cutting performance in FFF-printed thermoplastics using machine learning algorithms. Proc. Inst. Mech. Eng. Part E J. Process Mech. Eng..

[37] Der O. (2025). Multi-Output Prediction and Optimization of CO_2_ Laser Cutting Quality in FFF-Printed ASA Thermoplastics Using Machine Learning Approaches. Polymers.

[38] Mirinejad H., Parvinian B., Ricks M., Zhang Y., Weininger S., Hahn J.-O., Scully C.G. (2019). Evaluation of fluid resuscitation control algorithms via a hardware-in-the-loop test bed. IEEE Trans. Biomed. Eng..

[39] Varvel J.R., Donoho D.L., Shafer S.L. (1992). Measuring the predictive performance of computer-controlled infusion pumps. J. Pharmacokinet. Biopharm..

[40] Berard D., Vega S.J., Avital G., Snider E.J. (2022). Dual input fuzzy logic controllers for closed loop hemorrhagic shock resuscitation. Processes.

[41] Master T., Mark T. (2012). Medical Electrical Equipment Part 1: General Requirements for Basic Safety and Essential Performance.

[42] Marques N.R., Ford B.J., Khan M.N., Kinsky M., Deyo D.J., Mileski W.J., Ying H., Kramer G.C. (2017). Automated closed-loop resuscitation of multiple hemorrhages: A comparison between fuzzy logic and decision table controllers in a sheep model. Disaster Mil. Med..

[43] Snider E.J., Berard D., Vega S.J., Avital G., Boice E.N. (2022). Evaluation of a proportional–integral–derivative controller for hemorrhage resuscitation using a hardware-in-loop test platform. J. Pers. Med..

[44] Fage N., Asfar P., Radermacher P., Demiselle J. (2023). Norepinephrine and Vasopressin in Hemorrhagic Shock: A Focus on Renal Hemodynamics. Int. J. Mol. Sci..

[45] Smith J.B., Pittet J.-F., Pierce A. (2014). Hypotensive Resuscitation. Curr. Anesthesiol. Rep..

[46] Kowalenko T., Stern S., Dronen S., Wang X. (1992). Improved outcome with hypotensive resuscitation of uncontrolled hemorrhagic shock in a swine model. J. Trauma Acute Care Surg..

[47] Chalkias A. (2023). Shear Stress and Endothelial Mechanotransduction in Trauma Patients with Hemorrhagic Shock: Hidden Coagulopathy Pathways and Novel Therapeutic Strategies. Int. J. Mol. Sci..

[48] Gutierrez G., Reines H.D., Wulf-Gutierrez M.E. (2004). Clinical review: Hemorrhagic shock. Crit. Care.

